# Editorial: Materials for electroanalysis and electrocatalysis based on advanced frameworks

**DOI:** 10.3389/fchem.2022.1091608

**Published:** 2022-11-18

**Authors:** Chunying Xu, Baiqing Yuan, Dong Liu

**Affiliations:** ^1^ School of Chemistry and Materials Science, Ludong University, Yantai, China; ^2^ School of Agricultural Engineering, Jiangsu University, Zhenjiang, China

**Keywords:** metal-organic frameworks (MOFs), covalent organic frameworks (COFs), electrochemical sensors, electrocatalysis, oxygen reduction reaction (ORR), oxygen evolution reaction (OER), hydrogen evolution reaction (HER), carbon dioxide reduction reaction (CO2RR)

Reticular chemistry opened a new way to built crystalline structures material with the linking of molecular building units by strong bonds, which is developing chemistry beyond molecules (Yaghi, 2019). The extended structures such as metal–organic frameworks (MOFs) and covalent organic frameworks (COFs), is currently one of the most rapidly growing fields of science. MOFs are uniquely constructed by combining both inorganic and organic components from chelating linkages between metal ions/clusters and organic linkers (Li et al., 2021). COFs are two-dimensional (2D) and three-dimensional (3D) organic frameworks composed of light elements (e.g., B, C, N, and O) through strong covalent bonds, which extend organic chemistry beyond molecules and polymers.

Compared with the traditional porous materials such as zeolites, MOFs and COFs exhibit many special properties including large surface area, high porosity, tunable pore structures and ease functionalization. MOFs also show diverse composition and well-defined metal centers. They have been recognized as promising candidates in catalysis, separation, sensing, and biomedicine (Sun et al., 2021). In comparison with MOFs, COFs have much higher thermal and chemical stabilities due to the involvement of covalent bonds. MOFs and COFs have also been seen as attractive precursors to fabricate functional porous derivatives with precisely controlled compositions, structures, sizes and shapes by pyrolysis under an inert atmosphere. The porous derivatives include carbons, metal nanoparticles, metal oxides, metal phosphides, metal chalcogenides, metal carbides, and so on. Especially, COFs show superior properties for the production of derivatives because the strong covalent bonds in COFs, instead of metal ions or clusters coordinated to organic ligands in MOFs, construct very stable ordered structures with designable pore size, flexible molecular structure, and permanent porosity. COFs have exceptional thermal stabilities due to their covalent bond connection while MOF materials are unstable and easy to collapse at high temperature. Although many MOFs are prone to structural destruction even in an ambient atmospheric and water environment due to the lability of coordination bonds between metal ions and ligands, great efforts have been made in developing methods to improve the stability of MOFs including preparation of high-valent metalcarboxylate or low-valent metal-azolate MOFs, use of azole containing carboxylate linkers, mixed metal ions, and hydrophobic ligands, insertion of building blocks, framework interpenetration, etc. (Ding et al., 2019). In addition, post-synthetic structural processing and composite material engineering have also been explored to improve the stability of existing MOFs.

Pristine MOFs and COFs are also used to immobilize catalytically active materials or molecules into/onto the pores, matrices, or layers to improve the properties (Yang et al., 2021). The unique properties have made MOFs and COFs promising candidates in electroanalysis and electrocatalysis due to the following advantages. 1) The porous structure and high surface area facilitates the exposure of more active sites, increase the electrochemically active surface area (ECSA), allowing for rapid mass transport and electron transfer. 2) MOFs possess abundant metal active sites or redox active ligands which shows high electrocatalytical activity (Yuan et al., 2014; Zhang et al., 2015). 3) MOFs and COFs can also act as porous matrices to increase enrichment and adsorption ability for analytes to achieve sensitive detection of environmental pollutants. 4) Their tunable porosity endow them the ability to identify target analytes specifically through size selectivity effects (Fu et al., 2023). 5) MOFs and COFs based porous materials including porous derivatives can be used to immobilize biocatalytic components such as enzymes, proteins, redox molecules, and microbes for efficient electrochemical biosensors. They provide protection and nanoconfinement effects, or simply be used as supporting matrices to enhance the loading of biocatalytic components (Auer et al., 2022).

Although great progress has been made in electroanalysis, many challenges remain. Reversibility of the chemical reactions between ligands is an essential prerequisite to create the self-healing ability to repair structural defects in order to ensure the growth of the COFs crystalline structures (Huang et al., 2016). However, the insufficient self-healing process results in abundant defects in COFs, and the low molecular conjugation of π-electrons also causes electron localization, which leads to the intrinsic poor conductivity of bulk COFs and consequently limits their application in electrochemical sensing. This challenge can be resolved *via* different strategies including doping with oxidants and guest molecules, template synthesis, introducing conductive polymers, π-conjugated planar 2D structures, and the metalation of COFs (Chen et al., 2020). The poor conductivity of MOFs is also one of the biggest barriers to achieve sensitive electroanalysis. Efforts have been undertaken so far to overcome the problem by four strategies including the “through-bond” approach using continuous chains of coordination bonds, the “extended conjugation” approach forming large delocalized systems, “through-space” approach harnessing the π−π stacking interactions, and the “guest-promoted” approach utilizing the inherent porosity of MOFs and host−guest interactions (Xie et al., 2020). In addition, they can also serve as powerful supporter to achieve the combination with conducting materials such as graphenes, carbon nanotubes (CNTs), and metal oxide NPs, enhancing their electrocatalytic abilities and electrical conductivity.

The second challenge is the modification of materials on different electrodes. Most MOFs are rigid and focused on macroscaled crystalline products with hundreds of micrometers, which is not beneficial for the modification of electrode, reproducibility, and electrocatalytical response. The immobilization methods and strategies for COFs and MOFs on different substrates include solvothermal growth/deposition, electrophoretic deposition, electrochemical deposition, interfacial polymerization, and drop-coating. Ultrathin and nanoscaled MOFs and COFs based materials are attracting more and more attention to achieve easy modification and high electrocatalytic activity. Large size COFs and MOFs lead to low active area, low mass transfer rate, as well as poor stability on the electrode, which will influence the stability, reproducibility/repeatability, and sensitivity.

The third challenge is concerned with the antifouling capability which refers to the chemical fouling caused by the absorption of electrochemical product and biofouling due to the non-specific absorption of biological macromolecules for the analysis of biological fluid. However, the antifouling properties of MOFs and COFs have received comparatively little attention until recently. Design and fabrication of hydrophilic interface would be a strategy to solve the problem.

The fourth challenge is the real ECSA. Although MOFs and COFs have large surface area and high porosity, the area may not be fully utilized because solution may not enter into all the pores due to the hydrophobicity and microbubbles formed on the surface (Sun et al., 2022). The research about ECSA of MOFs and COFs is rare reported. Although many characterization methods of ECSA have been reported, new characterization and hydrophobicity evaluation methods on microscale are urgently needed.

The fifth challenge is linked with the fabrication of microelectrode based on MOFs and COFs or growth of these frameworks on microelectrode for *in vivo* and intracellular analysis, which offer efficient tool to better understand physiological or pathologic processes in central nervous system and sophisticated and mysterious biological processes in cells.

Benefiting from the unique properties, MOFs and COFs based materials have also shown potential in the field of clean energy conversion which is considered as a promising solution to the challenges of environmental protection and energy supplies caused by the sustainable growth consumption of fossil fuels. Oxygen reduction reaction (ORR), nitrogen reduction reaction (NRR), carbon dioxide reduction reaction (CO2RR), oxygen evolution reaction (OER), and hydrogen evolution reaction (HER), are five major reactions in the progress, conversion, and storage of clean energy. Efficient electrocatalysts can decrease the overpotential of these reactions and elevate practical applications. With the rapid development of nanotechnology, the synthesis and application of micro/nano-scaled MOFs and COFs in this field is receiving more and more attention. Micro/nanoframeworks can not only remain their original inherent characteristics, but also generate a series of unexpected physical and chemical performances through the nano-effects (Wei et al., 2022). In addition, 2D MOFs and COFs nanostructures can offer long-term stability, fast ion diffusion ability, and high electrical conductivity to enhance catalyst activity and durability. However, the 2D and conductive MOFs and COFs are synthesized with extremely expensive ligands, hindering the further explorations and applications for energy conversion (Khan et al., 2022). Therefore, it is of significance to explore the environmentally friendly and low-cost ligands to prepare these materials.

In this Research Topic, Rademacher et al. reported an efficient electrocatalyst based on iridium oxide (IrO_x_-NP) and palladium nanoparticles (Pd-NP) supported on a 2,6-dicyanopyridine-based covalent-triazine framework (DCP-CTF) by energy-saving and sustainable microwave-assisted thermal decomposition reactions in propylene carbonate and in the ionic liquid [BMIm][NTf_2_]. The composites material demonstrated superior performance toward HER with low overpotentials from 47 to 325 mV and toward ORR with high half-wave potentials between 810 and 872 mV, which lead to competitive electrocatalytic activities toward HER and ORR compared to commercial Pt^20^/C. Ahmed Malik et al. synthesized a series of Ce-MOF, GO@Ce-MOF, calcinated Ce-MOF, and calcinated GO@Ce-MOF used as high-proficient electrocatalysts for OER. The CeO_2_ immobilized in the MOF derived carbon showed high electrocatalytic activity and the introduction of GO improved the conductivity. Fan et al. reviewed the pioneering works in COFs-based materials for electrocatalytic CO2RR in recent years and provides a basis for future design and synthesis of highly active and selective COF-based electrocatalysts in this direction. Ehzari and Safari presented a highly accurate and precise sandwich-type electrochemical immunosensor for the electrochemical detection of human epidermal growth factor receptor 2 (HER2) based on the combination of Magnetic framework (Fe3O4@ TMU-24) and Au nanoparticles (AuNPs). The composites exhibited high specific surface area, excellent biocompatibility, excellent electrocatalytic properties, and powerful loading capability for HER2 antibody, which offered a wide linear range and low detection limit for the detection of HER2. Jiang et al. prepared a Pt-coordinated titanium-based porphyrin metal organic framework (Ti-MOF-Pt) by embedding single-atom Pt through strong interactions between the four pyrrole nitrogen atoms in the rigid backbone of the porphyrin and used to fabricate electrochemical aptamer sensor for the signal amplification based on the large surface area of MOFs. The aptamer sensor showed high selectivity and sensitivity for the detection of thrombin.

Although great progress has been made in the fields of electroanalysis and electrocatalysis, MOFs- and COFs-based materials show many foreseeable challenges that should be emphasized and well addressed in the future. For example, frameworks with high stability in the basic/acidic conditions and high electrical conductivity, and fabrication of low cost ligands will promote their widespread application in electroanalysis and electrocatalysis. Finally, we would like to thank all the authors for their contributions to this Research Topic. We also appreciate the referees and the editorial staff of Frontiers in Chemistry for their works in publication of this Research Topic.
